# Engineering and Applying Quantum Contextuality

**DOI:** 10.3390/e28040446

**Published:** 2026-04-14

**Authors:** Mladen Pavičić

**Affiliations:** 1 Center of Excellence for Advanced Materials and Sensors, Institute Ruder Bošković, 10000 Zagreb, Croatia; mpavicic@irb.hr; 2 Institute of Physics, 10000 Zagreb, Croatia

**Keywords:** quantum contextuality, hypergraph contextuality, MMP hypergraphs, MMP language, Kochen–Specker sets, non-Kochen–Specker contextual sets, quantum contextuality applications

## Abstract

The endeavor to refute hidden variable theories underlying quantum theory has yielded the discipline of contextual sets. A plethora of various kinds of sets of arbitrary structure in any dimension have been developed, alongside extensive experimental validation. These advancements incited us to investigate to what extent we might move beyond hidden variables, engineer contextual sets, and find their applications within quantum theory itself, without any reference to hidden variable models. To this end, we consider possible applications of contextual sets in quantum computation, cryptography, pseudo-telepathy, and nonlocality, as well as generating them from error-correction protocols, complex Hadamard gates, and simple quantum gates. We found that the results in this field are still scarce, and we therefore investigated the directions in which future research might be carried out and the potential obstacles to realizing such undertakings in the past.

## 1. Introduction

Quantum contextuality is a property of a set (let us call it a *contextual set*) of quantum states that precludes assignments of predetermined 0–1 values to their elements.

This feature of contextual sets has historically stemmed from procedures aiming to refute the existence of hidden classical variables underlying the quantum description of quantum systems, and it has so far mostly served us as a tool for distinguishing between quantum and classical sets. Along this line, experiments carried out so far [1,2,3,4,5,6,7,8,9,10,11,12,13,14,15,16,17,18,19,20,21] just confirm that implemented contextual sets really are contextual.

In contrast, we shall focus on engineering contextual sets and their applications without any reference to possible hidden variable models. In particular, we shall explore how quantum contextuality emerges from requirements we might expect quantum systems to possess and how we can find their application in quantum computation and networking.

In doing so, we shall not focus on how to generate new contextual sets by different methods but to what extent quantum theory and quantum measurements themselves generate contextual sets and whether there are instances of, say, quantum computation and quantum measurements that generate none but a contextual set or are generated by none but contextual sets. For instance, a recent analysis of stabilizer operations characterizing quantum error correction and therefore quantum computation [22] received the following review: ‘What gives quantum computers that extra oomph over their classical digital counterparts? An intrinsic, measurable aspect of quantum mechanics called contextuality, it now emerges’ [23]. But the question of ‘which contextualities’ has not been properly addressed so far.

In order to be able to speak about contextual sets, we first have to introduce the language that might describe and handle them. Its syntax will enable us to carry out our elaboration of cases as well as to identify failures when we fail to adhere to it.

The language we shall make use of is the language of MMP hypergraphs (MMPHs). There are other formulations, but most of them can be translated to MMPHs [24]. For the ones that cannot, see Section 2.4.

### 1.1. General Hypergraphs

A general hypergraph H=(V,E) is a set of points/vertices $V=\{v_{1},v_{2},\ldots ,v_{k}\}$ and a set of subsets/hyperedges $E=\{e_{1},e_{2},\ldots ,e_{l}\}$ of them [25,26,27,28]. The cardinality of *V* (|V|) (number of vertices) is called the *order* of a hypergraph, and the cardinality of *E* (|E|) (number of hyperedges) is called the *size* of a hypergraph.

### 1.2. MMP Hypergraphs and Their Language

A special kind of general hypergraph on which the formalism of contextual sets is founded is the McKay–Megill–Pavičić hypergraph (MMPH).

**Definition** **1.** **MMPH-dimension** *n is a predefined (for an assumed task or purpose) maximal possible number (n) of vertices within a hyperedge of an MMPH even when none of the processed hyperedges includes n vertices.*

**Definition** **2.** *An* **MMPH** *is a connected* hypergraph *H=(V,E) (where $V=\{V_{1},V_{2},\ldots ,V_{k}\}$ is a set of* vertices *and $E=\{E_{1},E_{2},\ldots ,E_{l}\}$ sets of* hyperedges*) of* MMPH-dim *n≥3 in which*

*1.*  *Every vertex belongs to at least one hyperedge.*
*2.*  *Every hyperedge contains at least 2 and at most n vertices.*
*3.*  *No hyperedge shares only one vertex with another hyperedge.*
*4.*  *Hyperedges may intersect each other in at most n−2 vertices.*
*5.*  *Graphically, vertices are represented as dots and hyperedges as lines passing through them.*

Numerically, an MMPH is a string of ASCII characters (vertices) organized in substrings (hyperedges) separated by commas. Each string ends with a period; when 90 characters 1…9 A…Z a…z ! " # $ % & ’ ( ) * - / : ; < = > ? @ [ ∖ ] _ ‘ { | } ∼; are exhausted, one reuses them prefixed by ‘+’, then by ‘++’, etc.—there is no limit.

Differences between the standard hypergraph and the MMPH formalism are illustrated in Figure 1 in [29].

**Definition** **3.** *A k-l* MMPH *of dim n≥3 with k vertices and l hyperedges, whose i-th hyperedge contains κ(i) vertices (2≤κ(i)≤n, i=1,…,l) to which it is* impossible *to assign* 1*s and* 0*s in such a way that the following* rules *hold*

*(i)*  * No two vertices within any of its hyperedges may both be assigned the value* 1*.*
*(ii)*  *In any of its hyperedges, not all vertices may be assigned the value* 0*.*

*is called a* non-binary MMPH *(***NBMMPH***).*

**Definition** **4.** *A k-l* MMPH *of dim n≥3 with k vertices and l hyperedges, whose i-th hyperedge contains κ(i) vertices (2≤κ(i)≤n, i=1,…,l) to which it is* possible *to assign* 1*s and* 0*s in such a way that the following* rules *hold*

*(i)*  * No two vertices within any of its hyperedges may both be assigned the value* 1*.*
*(ii)*  *In any of its hyperedges, not all vertices may be assigned the value* 0*.*

*is called a* binary MMPH *(***BMMPH***).*

**Definition** **5.** *A* **Critical NBMMPH** *is an* NBMMPH *which is minimal in the sense that removing any of its hyperedges turns it into a BMMPH.*

**Definition** **6.** **Vertex multiplicity** *is the number of hyperedges vertex ‘i’ belongs to; we denote it by m(i).*

**Definition** **7.** *A* **filled** *n-dim* MMPH *is the one that is derived from an n-dim* MMPH *with at least one hyperedge containing fewer than n vertices by adding vertices to ensure all hyperedges have precisely n vertices.*

### 1.3. Coordinatization

Vector or state or operator representation, i.e., a *coordinatization* of vertices is operationally required for any implementation of an MMPH since a full coordinatization of vertices turns MMPH-dimension *n* into a dimension of a Hilbert space determined by vectors each vertex is assigned to.

Our algorithms and programs can detect the contextuality of an MMPH no matter whether its coordinatization is given (or even existent) or not. Handling of MMPHs using our algorithms embedded in programs Shortd, Mmpstrip, One, Mmpsubgraph, Vecfind, States01, and others [29] without taking their coordinatization into account gives us a computational advantage over handling them with a coordinatization because processing bare hypergraphs is faster than processing them with vectors assigned to their vertices.

**Lemma** **1.** *An n-dim* NBMMPH *from Definition 3, with all hyperedges containing n vertices, which possesses a coordinatization is a* **Kochen–Specker (KS)** NBMMPH.

**Proof.**  Obvious [29,30,31,32].   □

**Lemma** **2.** *An n-dim* NBMMPH *from Definition 3, with at least one hyperedge containing fewer than n vertices, which possesses a coordinatization is a* **non-Kochen–Specker (non-KS)** NBMMPH.

**Proof.**  Obvious [29,30,31,32].   □

**Figure 1:**
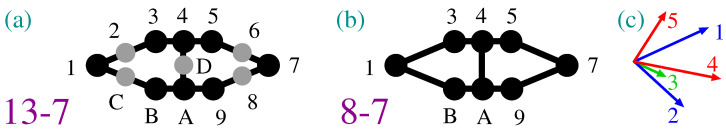
(**a**) Noncontextual 13-7 BMMPH—filled 8-7; (**b**) contextual 8-7 non-KS NBMMPH—unfilled 13-7; (**c**) Mathematica rotation of 12 to 45 around 3.

Notice that added vertices in filled MMPHs actually reveal hidden vectors that exist in the vector space due to vector orthogonalities and the structure of each MMPH and its hyperedges. More specifically, two adjacent *n*-dimensional hyperedges map to each other by rotation around an (n−2)-dimensional subspace. In a 3D space that means around an 1D vector. So, let us have a look at Figure 1.

The 13-7 MMPH is a noncontextual BMMPH. Its coordinatization is as follows.

**13-7** 123,345,567,789,9AB,BC1,4DA. 1 = $\frac{1}{\sqrt{3}}$(1,1,1), 2 = $\frac{1}{\sqrt{6}}$(2,−1,−1), 3 = $\frac{1}{\sqrt{2}}$(0,1,−1), 4 = (1,0,0), 5 = $\frac{1}{\sqrt{2}}$(0,1,1), 6 = $\frac{1}{\sqrt{6}}$(2,−1,1), 7 = $\frac{1}{\sqrt{3}}$(1,1,−1), 8 = $\frac{1}{\sqrt{6}}$(−1,2,1), 9 = $\frac{1}{\sqrt{2}}$(1,0,1), A = (0,1,0), B = $\frac{1}{\sqrt{2}}$(1,0,−1), C = $\frac{1}{\sqrt{6}}$(−1,2,−1), D = (0,0,1).

One arrives at the hyperedge 345 by rotating 12 (which spans the 2D space to which vector 3 is perpendicular) around vectors 3 to 45 (which lie in the same 2D space and which also span it). When we strip the gray vertices 2,6,8,C,D we arrive at 8-7 non-KS contextual NBMMPH which can serve us for particular purpose when elaborating on a chosen set of states but we always have to take the ‘hidden’ vectors into account. Take, e.g., an experiment with a spin-1 Stern–Gerlach device. The probability of an atom exiting from each of the three ports of a gate is 1/3. When we disregard measurement data from one of the ports, say from 2, we spoil the statistics since we discard 1/3 of the obtained data. Hence, we have to increase the measurement rate to reach the 1/2 probability of detection for 1 and 2 with respect to detections from all three ports at another gate. Also, if we decide to fill 8-7 so that all hyperedges have 3 vertices, this filling will by no means be arbitrary or random. The added vertices will be determined by the orthogonality requirements of vectors in each triple. Also, we can only determine a coordinatization of 8-7 by reading it off the coordinatization of 13-7; cf. Figure 5b in [33] and the text below. Also, if we try to arrive at 345 by rotating 1 to 45 in the 8-7 non-KS NBMMPH around vector 3, we would fail because there is no vector in the hyperedge 13 to be rotated to vector 4.

The paper is organized as follows.

In Section 2.2 we consider the misconceptions of measuring states that define structures of quantum contextuality. States are organized in sets of orthogonalities which form hyperedges of MMPHs and ultimately the MMPHs themselves. Of those states we might decide to measure some and not the others (non-KS NBMMPHs), but only all geometrically possible states define the structure of an MMPH (KS or noncontextual). We show that this is overlooked by many researchers in the field, which results in objectionable approaches to systems. In particular, we show that well-known 3D non-KS NBMMPHs cannot be minimal since none of them is critical and they all contain many smaller NBMMPHs. The same applies to the 4D stabilizer + magic state non-KS NBMMPH, which finds its application in quantum computation.

In Section 2.3 we consider a fusion of entanglement and nonlocality called the pseudo-telepathy game. Such games were originally conceived as another way of disproving hidden variable theories. We analyze some recent approaches to see whether they offer us a richer method of such a verification. In particular, whether the complete bases set up limits on it. We find that their role is richer than anticipated.

In Section 2.4 we consider the quantum shallow circuit advantage which stems from quantum nonlocality, which can be converted to quantum contextuality.

In Section 2.5 we point out that noncontextuality inequalities just serve the purpose of verifying whether a set is contextual although not always in a reliable manner.

In Section 2.6 we consider cryptography supported by MMPHs. They offer vast support and protection to large alphabet protocols. We show that what protects the protocols is the vast MMPH data base and not the contextuality of MMPHs.

In Section 2.7 a generation of star-like non-KS NBMMPHs from generalized complex Hadamard matrices is presented, and a way of obtaining coordination for low number of complete bases is elaborated.

In Section 2.8 a recent attempt to construct new large 3D sets and use them to obtain a lower bound on size of non-KS contextual sets is analyzed, along with an explanation for its failure.

In Section 2.9 we consider how often we stumble upon contextuality in random quantum setups and measurements, i.e., how ‘typical’ contextuality is.

In Section 3 we discuss the obtained results.

Methods used to yield the results obtained in the paper are presented in Section 4.

A conclusion is given in Section 5.

## 2. Results

In our previous papers we have shown how one can generate NBMMPHs of arbitrary structure and complexity in any dimension [24,29,34,35,36].

Here, we shall investigate whether one can obtain NBMMPHs from quantum artifacts (models, structures, and/or measurements) or make use of them to arrive at possible quantum applications without a reference to any hidden variable theory. While doing so, we shall highlight some misconceptions and misinterpretations that might stand in the way of a successful elaboration toward such a goal.

### 2.1. Quantum Engineering of Contextual Sets and Their Quantum Application

Contextual sets generated by the aforementioned quantum artifacts and applied to quantum systems subjected to quantum measurements without any reference to classical measurements can be framed by the following two formal definitions.

**Definition** **8.** *Quantum engineering of contextual sets is a procedure of their generation from a mathematical description of a set of possible or actual quantum measurements without any reference to a counterfactual or actual classical measurements with assumed predetermined set of outcomes.*

**Definition** **9.** *Quantum application of contextual sets is their putting to practical use in manipulating quantum systems without any reference to a counterfactual or actual classical setups with predetermined sets of measurement outcomes.*

### 2.2. Minimal KS Sets and Quantum Computation

Recently, a role of quantum contextuality in quantum error correction algorithms which made use of stabilizer states has been revealed [22], and its contribution to speeding-up quantum computation has been considered [37].

In doing so, graphs and hypergraphs were employed as prevalent tools. But before we dwell on the 4D stabilizer sets let us first discuss some relevant features of simple 3D hypergraphs/MMPHs in order to flesh out our points on handling the stabilizers.

**Figure 2:**
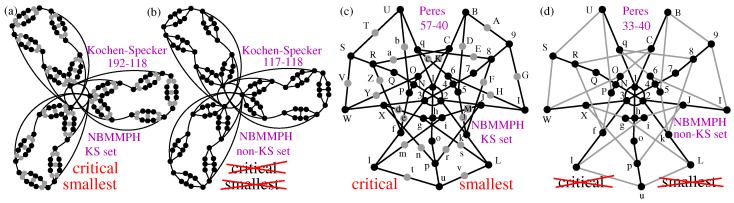
(**a**) Critical KS NBMMPH 192-118 according to [33] (Figure 6); its coordinatization is given for the first time in [35] (Supplemental Material, pp. 3–4). (**b**) Redrawn $\Gamma_{2}$ [30] following Figures 6 and 8 from [40]; it is not critical and therefore also not the smallest in its class; see Appendix A. (**c**) Peres’ 57-40; vertices with multiplicity 1 (gray dots) are added to [41] (Figure 9); it has a 81-52 master. (**d**) Peres’ 33-40 according to Figure 9 from [41]; it is also neither critical nor the smallest in its 81-52 class; see Appendix A.

There is a widespread misconception that the historic contextual sets 33-36 Bob-Schütte, 33-40 Peres, 31-37 Conway-Kochen, 117-118 Kochen–Specker, and 33-50 Pavičić sets are the smallest possible 3D contextual sets, two of which are shown in Figure 2b,d. However, being the smallest set means being a critical set in the sense that dropping any of the edges of such a set renders it non-contextual. Hence, since they are claimed to be the smallest, they should not contain contextual sets smaller than themselves. Yet, none of the aforementioned five 3D sets is critical and they all contain a large number of smaller critical non-KS contextual sets [38,39].

The sets that are genuine representatives of the smallest 3D KS sets are those with all vertices in all hyperedges (vector triples): 49-36 Bob-Schütte, 57-40 Peres, 51-37 Conway-Kochen, 192-118 Kochen–Specker and (complex) 69-50 Pavičić’s set [29] (Figure 10e, string on p. 55) since they are all critical. Two of them are shown in Figure 2a,c.

The misconception about non-KS contextual vs. KS contextual sets might have originated from historical statements such as Peres’: ‘It can be shown that if a single ray is deleted from that set of 33, the contradiction disappears’ [42] (p. 199), which is wrong, as our program STATES01 shows [38] (p. 7). Further details are given in Appendix A.

So, when we deal with a non-KS NBMMPH, in order to verify whether it possesses a particular feature, e.g., whether it might have a coordinatization, we have to first fill it up (add all missing vertices with multiplicity 1) and then check whether the filled MMPH has a coordinatization.

And vice versa, when we want to find a specified non-KS NBMMPH among subsets of a given MMPH we just strip it of vertices and check it on the non-KS feature. For example, when stripping edges and vertices off Peres’ KS 57-40 we might arrive at Yu and Oh’s non-KS NBMMPH 13-16 [43], i.e., its filled MMPH 25-16 (non-contextual) is a subhypergraph of the 57-40.

We encounter both options when we encounter contextuality within quantum computation [22,44].

It is considered that quantum computation with qubits is most efficient when stabilizer circuits constructed via Clifford gates (Hadamard, c-NOT, and phase gate) are used for preparing qubits (in stabilizer states), which are then combined with ancillary qubits in *magic* states. Qubits in the former states cannot be used for quantum speed-up since they may be simulated on a classical computer (Gottesman–Knill theorem [44]). But their combination with ancillary qubits in the latter states can and a choice of their combined states proves to be contextual [22,45]. In particular, graph Γ (30-108) shown in Figure 2 in [22] in which the vertices correspond to two-qubit stabilizer states and their orthogonalities/hyperedges correspond to the effect of ancillary qubits in magic states on them proves to be contextual.

**Figure 3:**
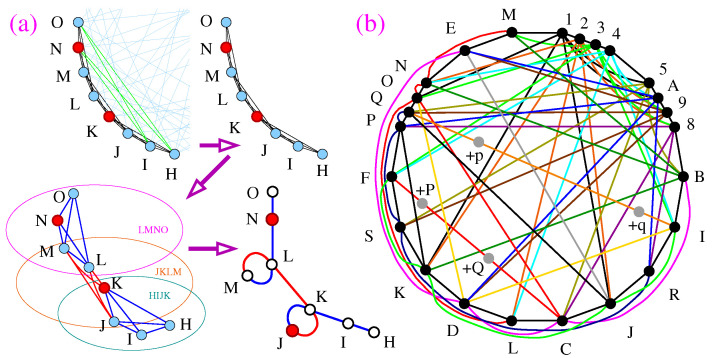
(**a**) A partial translation of a graph representation of several connected cliques from the 30-108 Γ graph from [22] (Figure 2) into a hypergraph representation of the corresponding hyperedges from the 30-108 non-KS NBMMPH from [29] (Figure 6a); (**b**) Critical KS subhypergraph (152-71 MMPH) of the 232-108 MMPH; its coordinatization is given in Appendix E and its ASCII string in Appendix E in [29]; not all vertices with multiplicity 1 (gray dots) are shown.

The graph 30-108 with 30 vertices and 108 hyperedges (30-108) is presented in the MMPH language and analyzed in [29] (Figure 10e, pp. 37, 38, 56). It is shown that its filled MMPH 232-108 is contextual and therefore that it is a genuine KS set in the same way as, e.g., Peres’ 57-40 above is, only it is itself not critical. It contains a single 152-71 critical subhypergraph which is shown in Figure 3b, which is the minimal critical MMPH generated from vector components {0,±1,±2,±3,5} as Peres’ 57-40 above is from $\left\{0,\pm 1,\pm \sqrt{2},3\right\}$.

The approach should be elaborated on contextuality of the operations supporting quantum speedup. Is there a simplified combination of stabilizer and magic states that corresponds to the 152-71 KS or 24-71 non-KS NBMMPH [45]? (We obtain 24-71 stripping 152-71 from all gray vertices.) Are there noncontextual operations that support quantum speedup? Which other operations support the speedup [46]?

Notice that MMPHs cannot be used for universal hypergraph representation of stabilizer codes [47] because the definition of MMPH restricts a general hypergraph; e.g., a hyperedge containing only one vertex is not allowed. In order to allow this and some other features, one should rewrite all MMPH algorithms and programs.

### 2.3. Pseudo-Telepathy Games

A fusion of entanglement, nonlocality and refutation of hidden variables has recently been formulated as a pseudo-telepathy game [48] (also known as a ‘bipartite perfect strategy’ [49]). When a state of one of two entangled particles is determined by a measurement we immediately know what a measurement of the state of the other particle would yield. We might be tempted to ascribe telepathic ability to particles, or to the measuring device, or to ourselves, or to Alice (owner of the 1st particle) over Bob (owner of the 2nd particle), but since the outcome of the measurement of the state of the first particle is completely random, the measurement of the first particle cannot serve Alice for transferring any information to Bob—so, ‘pseudo-telepathy.’

Inasmuch as a refutation of hidden variables is tightly supported by contextual sets, pseudo-telepathy has been used to grant Alice and Bob active roles in the so-called pseudo-telepathy games. The crux of, say, a two-player (A,B) game *G* of dimension *d* is that it does not have a classical winning strategy while it does have a quantum winning strategy (the players share a prior entangled state). Here a critic might ask why a classical wining strategy does not include a classical entangled state, but the answer might be that hidden variable theories do not assume it.

Regardless, under the former strategies the maximal, classical (*C*) vs. quantum (*Q*), winning probabilities ω satisfy the following inequality:

(1) $$\begin{array}{c} \omega_{C}\left(G\right)<\omega_{Q}\left(G\right)=1 \end{array}$$

A two-player game *G* is a tuple 〈X,Y,A,B,P,W〉, where *X*, *Y*, *A* and *B* are finite sets; P⊆X×Y and W⊆X×Y×A×B, where *X* and *Y* are the input sets; *A* and *B* are the output sets; *P* is a predicate on X×Y known as the *promise*; and W is the winning condition, which is a relation between inputs and outputs that has to be satisfied by Alice and Bob whenever the promise is fulfilled. Alice is asked a question x∈X, and she produces an answer a∈A. Bob is asked a question y∈Y, and he produces an answer b∈B. They are not allowed to communicate after they have received their questions. They win if (x,y,a,b)∈W. Their quantum winning strategy is that they share an entangled state:

(2) $$\begin{array}{c} \frac{1}{\sqrt{d}}\sum_{j=0}^{d-1}|j\rangle |j\rangle \end{array}$$

The KS game is defined relative to the above set of vectors. Alice receives a random *n*-tuple of orthogonal vectors as her input, and Bob receives a single vector randomly chosen from the *n*-tuple as his input. Alice outputs *d*-tuple indicating which of her *d* vectors is assigned color 1 (implicitly, the other n−1 vectors are assigned color 0). Bob outputs a bit assigning a color to his vector. The requirement is that Alice and Bob assign the same color to the vector that they receive in common.

The original pseudo-telepathy KS game goes as follows [48]. Alice is given only two vectors $v_{1}$ and $v_{2}$. She chooses d−2 additional vectors $v_{3},\ldots ,v_{d}$, so that $v_{1},\ldots ,v_{d}$ form an orthogonal *d*-tuple, performs a measurement on her share of the entangled state in basis $B_{a}=\{|v_{1}\rangle ,\ldots ,|v_{d}\rangle \}$ and obtains *k* as the result—she assigns 1 to vector $v_{k}$. Bob is given vector $v_{l}$. He chooses d−1 additional vectors $w_{1},\ldots ,w_{d-1}$ so that $v_{l},w_{1},\ldots ,w_{d-1}$ form an orthogonal *d*-tuple. The promise *P* states that $|v_{l}\rangle$ is from Alice’s basis. Bob performs a measurement on his share of the entangled state in basis $B_{b}=\{|v\rangle ,|w_{1}\rangle ,\ldots ,|w_{d-1}\rangle \}$ and obtains the output b=1 if the outcome is $|v_{l}\rangle$ and b=0 otherwise [48] (Equations (2.2), (3) and (4)).

In other words, the original pseudo-telepathy game is just another way of proving that a KS set really is a KS set. This is what our program States01 does in no time for any KS in any dimension. But what we need are applications which would contribute to proper quantum computation and not just refute a hidden variable theory.

Is a recent redefinition of a KS pseudo-telepathy game [49] on such a road (‘KS sets are crucial ingredients in some important results in quantum foundations and quantum computation’)? In the original pseudo-telepathy game the promise *P* states that Bob’s vector is orthogonal to a chosen Alice’s vector from Alice’s bases. Ref. [49] changes this so that the promise *P* states that Bob’s vector is *not orthogonal* to a chosen Alice’s vector and that Alice and Bob are each given a subset of vertices, *X* and *Y*, respectively; the pseudo-telepathy game is renamed *bipartite perfect quantum strategy* (BPQS) [49,50].

But to obtain an answer to our question on whether this redefined pseudo-telepathy game might contribute to quantum computation, we first have to address questions which the recent proposal left open.

The novel non-orthogonality pseudo-telepathy seems to trace the following reasoning. Two non-orthogonal vertices are disjoint (non-adjacent), i.e., there does not exist a hyperedge containing both of them. The maximum number of 1s one can assign to vertices satisfying the conditions (i) and (ii) from Definition 3 is therefore, by definition, a maximum number of pairwise disjoint vertices because all vertices belonging to a hyperedge to which only 0s can be assigned are adjacent to some vertices which are assigned value 1. This number is called the *independence (stability) number* and is denoted by α [27] (p. 151). Calculating α is an NP-complete problem which is solvable on a non-deterministic Turing machine [51]. (At any step the machine chooses from a set of possible moves; our 0-1 computers are deterministic.) It is related to a number denoted by $\alpha^{*}$, called a *fractional independence number*, and defined as the maximum value of $\sum_{v=1}^{k}x\left(v\right)$, where *v* is a vertex and x(v) a non-negative real number such that $\sum_{v\in e}x\left(v\right)\leq 1$ for each hyperedge *e*. The x(v) can be written as $w_{v}p\left(v\right)$, where p(v) is the probability of detecting the state of the vertex *v* corresponding to a system exiting through one of the ports of a gate corresponding to the hyperedge containing *v* and $w_{v}$ is its weight. Calculating $\alpha^{*}$ is also an NP problem.

The aforementioned relation is expressed via Equation (3a,b) inequalities.

(3a) $$\begin{array}{c} \mathrm{general}\;:\;\;\alpha \leq \alpha^{*};\;\left(?-\mathrm{IV}\;\mathrm{below}\right); \end{array}$$

(3b) $$\begin{array}{c} \mathrm{contextual}\;:\;\;\alpha <\alpha^{*}.\;\left(?-\mathrm{IV}\;\mathrm{below}\right) \end{array}$$

In the literature, Equation (3b) is taken to be a noncontextuality inequality, i.e., the one which verifies that an MMPH is contextual [22,52]. However, none of the two inequalities holds for standard quantum measurements—see III below.

**Figure 4:**
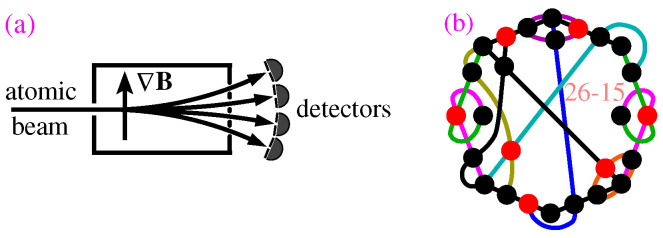
(**a**) Stern–Gerlach experiment with spin-$\frac{3}{2}$ atoms; the probability of each of the four detectors detecting an atom is $\frac{1}{4}$. (**b**) The 26-15 KS MMPH that violates Equation (3a,b); the vertices that contribute to the maximum number of 1s, i.e., to α=7 are colored in red; $\alpha^{*}=\frac{26}{4}=6.5$.

The two αs have the following properties relevant for pseudo-telepathy protocols.

(I) Individual quantum systems exiting any port of a gate of a standard quantum apparatus, e.g., in a (generalized) Stern–Gerlach experiment [53], shown in Figure 4a, have equal probability of being detected; therefore $w_{v}=1$ for all *v*s.
(II) When calculating α we can go around the NP-complete complexity by making use of our algorithms to get possible numbers of 1s by multitasking and obtain the maximal number, i.e., α, via saturation in polynomial time (P complexity). (Thousand + tasks in parallel are started on a supercomputer and stopped when no new numbers appear.)
(III) Assuming constant vertex probabilities at each gate/hyperedge for a filled MMPH (every hyperedge contains *n* vertices) $\alpha^{*}=\frac{k}{n}$, where *k* is the number of vertices in the MMPH (the probability of detecting the system at each port of a gate/hyperedge is $\frac{1}{n}$), for unfilled MMPHs one has to calculate probabilities separately for hyperedges containing fewer than *n* vertices [29].
(IV) For standard quantum apparatuses, both Equation (3a,b) are violated by arbitrarily many MMPHs. An example of such an MMPH is shown in Figure 4b. Others are given in [29] (Figures 6, 13b and 16b and Tables 2, 3 and 5).

The MMPH used in [49] for a pseudo-telepathy non-orthogonality protocol is contextual and does satisfy Equation (3b): $10=\alpha <\alpha^{*}=\frac{33}{3}=11$, but, in general, a contextual MMPH need not satisfy Equation (3b) and therefore it is not a noncontextuality inequality. Let us see how an MMPH can be handled without a reference to Equation (3b). Notice that Equation (3b) was used in the [45] protocol to prove its contextuality (Section 2.2).

**Figure 5:**
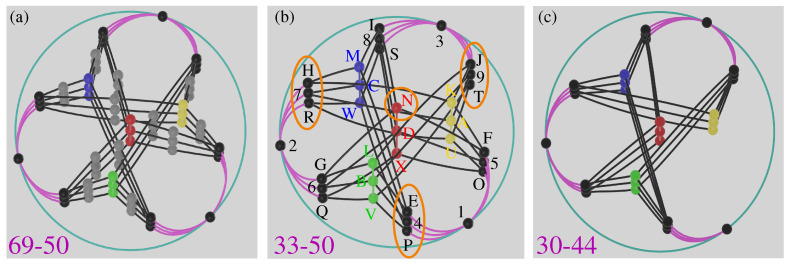
(**a**) Top snapshot of a 3D representation of the 69-50 set from a Blender output obtained in [54] which the reader can interactively rotate at will; (**b**) top snapshot of a 3D representation of the 33-50 set from a Blender output obtained in [55] which the reader can interactively rotate at will; vertices that show α=10 are encircled in orange; notice that three vertices in each orange encirclement are not mutually orthogonal—see Figure 6b; (**c**) top snapshot of a 3D representation of the 30-44 set from a Blender output obtained in [56] which the reader can interactively rotate at will.

**Figure 6:**
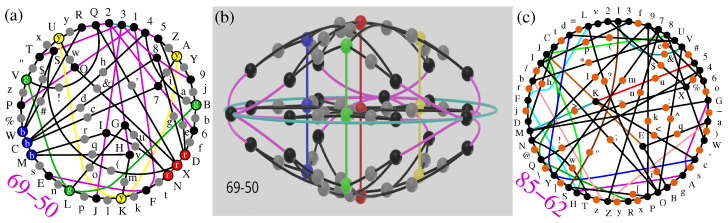
(**a**) The 69-50 MMPH from the 169-120 class [29] (Figure 10e) redrawn so as to emphasize colored vertices from [49] (Figure 1); its variety with gray vertices of multiplicity 1 dropped—33-50—is isomorphic to [49] (Figure 1) and to Figure 5b below; its coordinatization is given in the Appendix E. (**b**) A 3D representation (hypergraph dimensionality) of the 69-50 set; side snapshot from from a Blender output obtained in [54] which the reader can interactively rotate at will. (**c**) The 85-62 MMPH from 169 to 120 class with 15 complete bases; its coordinatization is given in Appendix E.

Within an MMPH, when implementing the non-orthogonality pseudo-telepathy protocol, we might have different distribution of vertices contributing to α=10, and therefore their balanced grouping in Alice and Bob’s bases has a great deal of symmetry illustrated by 69-50 MMPH in Figure 5b [29,34]. (Complex MMPHs exhibit more symmetries than real.) These symmetries serve Alice and Bob to obtain a balanced grouping of bases distributed in *X* and *Y*. They are established in the 3D MMPH-dimension (Definition 1) and are shown in Figure 5a and Figure 6a. If we wish to obtain its representation in a real vector space, we should do that in a 6D space. Ref. [49] (Figure 1) considers a 33-50 non-KS NBMMPH, obtainable from the 69-90 MMPH [29,34] by dropping the vertices with multiplicity 1. (By deleting the gray vertices from Figure 5a and Figure 6a,b we obtain Figure 5b.)

We see that we can carry out the aforementioned grouping by putting the vertices from the outer rim of the 33-50 (Figure 5b), i.e., (145,1EF,…) in *Y* and the ones from the inner rim (NDX,MCW,…) (red, yellow, green, blue) in *X*. The ‘new record’ 33-50 set of [49] (Figure 1) is isomorphic to our 33-50, and it is claimed in [49] that the set is the simplest because it contains 14 complete bases (hyperedges containing 3 vertices) while other 3D KS MMPHs contain 16 or more such hyperedges. (Here we just point out that the 85-62 shown in Figure 6c has 15 such hyperedges.)

**Figure 7:**
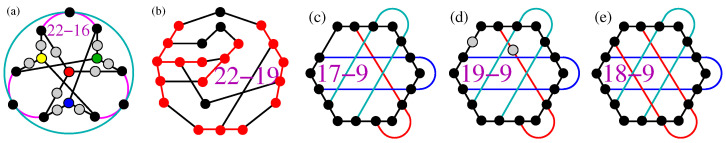
(**a**) Partially ‘extended’ Yu–Oh non-KS NBMMPH which is still contextual and has thirteen complete bases; (**b**) non-KS sub-NBMMPH of the 33-50 non-KS NBMMPH with seven complete bases; (**c**) a contextual critical non-KS sub-NBMMPH of the 18-9 KS MMPH obtained by means of a weak deletion of a vertex [27] (Section 7.4); (**d**) non-contextual *weakly extended* 17-9; (**e**) contextual *strongly extended* 17-9.

These ‘complete bases’ within a pseudo-telepathy 3D exclude hyperedges containing vertices with multiplicity 1 which apparently might be considered as ‘complete bases’ as well. For instance, Yu–Oh’s non-KS NBMMPH 13-16 [39] (Figure 1a) with nine vertices with multiplicity 1 added, so as to form the non-KS NBMMPH 22-16, has 13 such complete bases as shown in Figure 7a. Likewise, the non-KS NBMMPH 30-44 with 11 complete bases, shown in Figure 5c, might be considered as a legitimate candidate for a pseudo-telepathy game. From 33 to 50 we obtain the non-KS NBMMPH 22-19 with seven complete bases shown in Figure 7b. In 4D we can have candidates smaller than 18-9 for a pseudo-telepathy game such as 17-9 shown in Figure 7c.

On the other hand the MMPH requires orthogonal connections of groups (*X*, *Y*) of ‘complete bases’ via ‘incomplete’ bases, i.e., via hyperedges which contain fewer than *d* vertices, to establish a pseudo-telepathy game (e.g., ‘Alice and Bob win except when they output the two orthogonal vectors.’ [49] (p. 3, left column, 3rd par.)). This is because *X* and *Y* do not contain just non-orthogonal vertices (definition of α). The others, also contained in them, might be mutually orthogonal. When taking all combinations into account, Ref. [49] claims to have arrived at a graph stemming from 333 winning combinations of measurements which is not provided but for which it is claimed that α=44 holds.

To sum up, we analyzed a recently proposed non-orthogonality pseudo-telepathy game for the 33-50 non-KS NBMMPH to see whether it might contribute to a development of quantum computation. We obtain that the 33-50 is not the simplest/smallest non-KS NBMMPH since it is not critical and therefore contains many smaller non-KS sub-NBMMPHs, all of which contain a smaller number of complete bases which apparently might be used to establish pseudo-telepathy game protocols. The procedure of obtaining data for the protocols does not make use of any external data and is not applied to any quantum computation protocol. Hence, at least in the presented form, the proposed non-orthogonality pseudo-telepathy game is just another kind of disproving hidden variable theories. Here we have to bear in mind that such pseudo-telepathy games (with arbitrarily many suitable small and large, real and complex MMPHs) might be limited to disproving hidden variable theories so long as the standard quantum apparatuses are embraced, i.e., so long as equal probabilities of detecting systems through ports of gates/hyperedges are assumed. That means that if one finds quantum dynamics with variable probabilities at gate ports, the inequalities given by Equation (3a,b) would hold and might be employed to gauge, calibrate, and classify sets of quantum systems within quantum computation procedure. To verify whether such an approach is promising would require further investigation.

### 2.4. Contextuality vs. Nonlocality

Since the contextuality we consider can be converted into a corresponding nonlocality [57] and vice versa [58], an application of nonlocality is at the same time an application of contextuality, and the recent quantum advantage achieved with shallow circuits seems to be just that [59,60].

Classical shallow circuits are the ones of depth independent of the input size, where *depth* is the longest path in the directed acyclic graph where nodes are, e.g., AND or OR. Quantum shallow circuits are the ones with a usually constant number of layer depth (time steps) which does not scale with the number of qubits. The proven computational quantum advantage of quantum over classical shallow circuits is shown to be a consequence of quantum nonlocality [59]. In particular, within circuits with constant depth the correlations present in the measurement statistics of entangled quantum states exhibit quantum nonlocality. A proof is provided that this type of quantum nonlocality provides a relational problem that cannot be solved by general constant-depth classical circuits in the case when a nonlocal state is replaced by a graph state.

This result remains to be translated to the hypergraph language. We should also check on the universality of the result.

**Figure 8:**
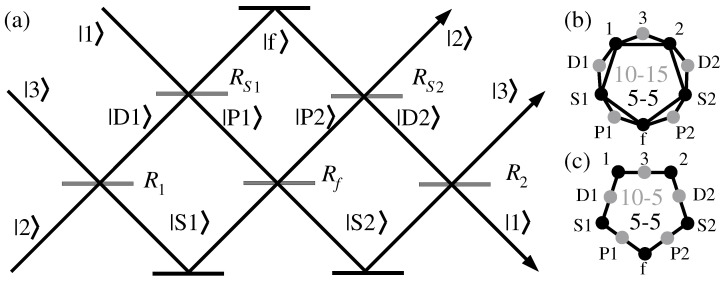
(**a**) Three-path interferometer according to [64] (Figure 1); reflectivities $R_{1}=R_{2}=1/2$, $R_{S1}=R_{S2}=1/3$, and $R_{f}=1/4$ maximize the contextual outcome; (**b**) graph of measurement results; each clique represents weak measurements related to a complete orthogonal basis [65]; (**c**) the same graph in the MMPH notation—Klyachko’s pentagon [29] (Figure 5; p. 16).

There is also a type of contextuality—Hardy-type [61], i.e., the logical contextuality [62]—and its corresponding nonlocality that does not always allow an MMPH representation. For instance, its simplest implementation with the entangled state

(4) $$\begin{array}{c} \Psi =\frac{1}{\sqrt{3}}(|00\rangle +|01\rangle +\left|11\rangle \right) \end{array}$$

does not have an MMPH representation [63]. However, its more elaborated implementations do have MMPH representations. One of them, a three-path interferometer implementation developed by Holger Hofmann [64,65,66], is shown in Figure 8a. 

In this setup the state given by Equation (4) exhibits contextuality [67]. It is obtained from the weak values of path projectors. ‘A weak value of a quantum variable …is a physical property of a quantum system between two measurements, i.e., a property of a system belonging to an ensemble that is both preselected and postselected …Due to this property weak measurements can be used as an amplification scheme.’ [68].

The weak measurements can be useful for several implementations apart from exhibiting quantum contextuality [69]. It would be important to contrast contextual with noncontextual setups with weak quantum states which are inaccessible by direct measurements to find out in which way they might differ, in particular because ‘weak values and their associated conditional currents provide a link between the wave-like propagation of quantum particles and their localized detection in only one of the output paths.’ [64].

### 2.5. Inequalities

Various inequalities which are and those which are not noncontextuality inequalities are analyzed and discussed in detail in [29]. In particular, it is shown that the so-called GLS inequality is violated by arbitrarily many MMPHs and that it is therefore not a noncontextuality inequality—see Figure 4b. Genuine noncontextuality inequalities serve for theoretical and/or experimental verification of contextuality of an MMPH and are therefore not ‘applications’ of quantum contextual sets.

### 2.6. Quantum Cryptography

In [70] a quantum cryptography protocol based on a KS scheme from [71] has been proposed and claimed to be ‘protected’ by KS contextuality. It is assumed that the KS architecture would give the protocol a quantum advantage. But does it?

Let us consider the following KS BB84-like protocol however not limited to four states (a pair of orthogonal states). Since over 100×100 entangled angular momentum dimensionality is experimentally achievable [72], the number of orthogonal pairs is practically unlimited. The protocol is classified as a large alphabet protocol [73].

- Alice sends outputs from gates/hyperedges of a chosen MMPH to Bob in blocks; she can repeat sending from the same MMPHs or pick up new ones.
- Bob stores Alice’s sending in a quantum memory (e.g., photons in fiber loops).
- Alice informs Bob about which sending belonged to which hyperedge over a classical channel (with a delay).
- Bob reads them off, scrambles them, and sends them back to her over the quantum channel; scrambling code unlocks Bob’s message (still unknown to Alice).
- Alice stores Bob’s sending into a quantum memory.
- Bob informs Alice of the scrambling code over a classical channel (with a delay).
- After an agreed number of exchanged blocks they can announce some messages over a classical channel to check whether Eve is in the quantum channel.
- After Alice correlated the reflected sending with the original ones via Bob’s code, she learns how to measure each of them from the quantum memory and read off Bob’s message.

A 4D example of the protocol is provided in [24] (pp. 20, 21). However, although MMPHs are used in the protocol and implementation, their KS contextual vs. classical noncontextual properties are not. Alice sends quantum state messages without juxtaposing them to any predetermined classical scheme. The only contraposition which might be encountered is with a classical predetermination-led eavesdropper (Eve). But Eve knows that Alice and Bob’s protocol is quantum and will therefore not base her interception on some predetermined hidden-variable model. Instead she will adopt the quantum gate balanced output procedure.

Hence, the KS large alphabet cryptography does offer higher security not because of contextuality but because it draws inputs from a vastly abundant source of MMPHs. Actually, the MMPHs can be non-contextual as well as KS or non-KS contextual. Briefly, the KS QKD protocols do not offer a quantum advantage over any other possible large alphabet protocols.

### 2.7. Generalized Complex Hadamard Matrices and Star-like Contextual MMPHs

Most known quantum computation algorithms are based on the the quantum Fourier transform, and the real Hadamard (*H*) transform is a special case of it [74], [75] (Section 3.3). When implemented, it takes the role of an *H*-gate within a quantum network. As we stressed above, it belongs to a collection of Clifford gates which themselves alone do not offer a quantum speed-up of the network. Whether a generalized complex *H*-transform can offer the speed-up seems to be an open question.

The real *H* matrix $H=[H_{ij}]$ is a matrix which satisfies the conditions: (1) $HH^{T}=nI_{n}$; (2) $|h_{ij}|=1$ for 1≤i,j≤n. That means that

(5) $$\begin{array}{c} H_{2}=\left[\begin{array}{cc} 1 & 1\\ 1 & -1 \end{array}\right],\;\mathrm{and}\;H_{4}=\left[\begin{array}{cc} H_{2} & H_{2}\\ H_{2} & -H_{2} \end{array}\right],\;\mathrm{and}\;\mathrm{recursively}\;\mathrm{constructed}\;\mathrm{matrices}\;\mathrm{of}\;\mathrm{order}\;2^{n} \end{array}$$

are all Hadamard matrices. Their order is even, but this recursive generalization does not yield matrices of all even orders, e.g., 6 and 10 are missing. (Note that the next *H* in Equation (5) is $H_{8}$ containing four $H_{4}$s.)

A complex generalization, on the other hand, seems to fill the gaps—it is conjectured (‘Complex Hadamard conjecture’ [76] (p. 68)) that it yields complex *H* matrices for any even order of *n*. So far, there are constructive proofs for even *n*s up to 70. The considered generalization is a *quaternary complex Hadamard matrix* (a kind of *Butson matrix* [76] (Section 4.1)) of even order *n* which is an n×n matrix with entries from {±1,±i} such that $HH^{*}=nI_{n}$, where * stands for conjugate transpose. This condition is a substitute for the condition (2) above. In particular we have

(6) $$\begin{array}{c} H_{c2}=\left[\begin{array}{cc} 1 & -i\\ 1 & i \end{array}\right],\;H_{c4}=\left[\begin{array}{cccc} 1 & 1 & 1 & 1\\ 1 & i & -1 & -i\\ 1 & -1 & 1 & -1\\ 1 & i & -1 & i \end{array}\right],\;H_{c6}=\left[\begin{array}{cccccc} 1 & 1 & 1 & 1 & 1 & 1\\ 1 & -1 & i & -i & -i & i\\ 1 & i & -1 & i & -i & -i\\ 1 & -i & i & -1 & i & -i\\ 1 & -i & -i & 1 & i & i\\ 1 & i & -i & -i & i & -1 \end{array}\right],\;\mathrm{etc}. \end{array}$$

Another kind of Butson Hadamard matrices which exists for other orders of *n* than the real Hadamard matrices is those with 1, ω, and $\omega^{2}$ ($\omega =e^{2\pi i/3}$ being the cube root of 1) as their elements [77] (p. 3). The two smallest of them are

(7) $$\begin{array}{c} \Omega_{3}=\left[\begin{array}{ccc} 1 & 1 & 1\\ 1 & \omega & \omega^{2}\\ 1 & \omega^{2} & \omega \end{array}\right] \end{array},\;\;\;\Omega_{6}=\left[\begin{array}{cccccc} 1 & 1 & 1 & 1 & 1 & 1\\ 1 & 1 & \omega & \omega & \omega^{2} & \omega^{2}\\ 1 & \omega & 1 & \omega^{2} & \omega & \omega^{2}\\ 1 & \omega & \omega^{2} & 1 & \omega^{2} & \omega \\ 1 & \omega^{2} & \omega & \omega^{2} & 1 & \omega \\ 1 & \omega^{2} & \omega^{2} & \omega & \omega & 1 \end{array}\right].$$

An analogous coordinatization of the smallest 6D star-like 21-7 KS MMPH [78] might have prompted Lisoněk to add a third *H* condition to the aforementioned two,

(8) $$\begin{array}{c} \forall \;k,l\;\left(1\leq k,l\leq n,\;k\neq l\right)\;\sum_{j=1}^{n}h_{kj}^{2}h_{lj}^{2}=0, \end{array}$$

thus arriving at what he calls *S*-*H* matrix [79] with elements including $\zeta_{g}=e^{2\pi i/g}$ (*g*-th root of 1), which he then uses to prove the following theorem.

**Theorem** **1.** *[79] Suppose that there exists an S-H matrix of order n (n even); then there exists a KS hypergraph k-l in $C^{n}$ such that k≤n+12 and l=n+1.*

From [78], it is not clear whether an *S*-*H* matrix for n=8 exists, but the star-like 8D 36-9 MMPH does exist, and one of its coordinatizations is real ({0,±1}) [80] while finding a coordinatization already for the star-like 10D 55-11 MMPH [36]; see Ref. [24] (Figure 13) is computationally too demanding. So, implementations of KS MMPHs with k=n+12 is presently highly unlikely.

However, a prospect of connecting generalized Hadamard gates with smaller non-KS NBMMPHs we might derive from the (2n−1)-(n+1) star-like KS MMPHs is intriguing since their coordinatizations are easily computable for higher dimensions. Notice that a deletion of all inner vertices of a star-like MMPH (leaving only its tip vertices) yields a non-KS NBMMPH. This explains why an *n*-gon (another form of a star [29] (Figure 5a,c)) is contextual for any even *n* and noncontextual for any odd *n*—the Schläfli symbol of a regular star is $\left[n+1/\frac{n}{2}\right]$ ($\frac{n}{2}$ has to be an integer). Hence, any contextual star-like MMPH between a non-KS NBMMPH (n+1)-(n+1) and a KS $\frac{n}{2}\left(n+1\right)$-(n+1) is critical.

**Figure 9:**
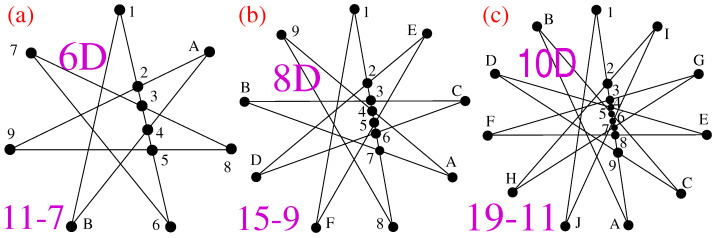
Star-like non-KS NBMMPHs (2n−1)-(n+1) with one complete hyperedge generated from KS contextual MMPHs $\frac{n}{2}\left(n+1\right)$-(n+1) obtained in [36]. (**a**) The smallest critical 6D non-KS NBMMPH 11-7, which, unlike its generator KS 21-7, allows a coordinatization from {0,±1} vector components; (**b**,**c**) also allow such coordinatizations. Strings and coordinatizations are given in Appendix E.

Recalling that the stabilizer MMPHs as well as the quantum game ones are all non-KS contextual, this option of obtaining non-KS critical NBMMPHs with arbitrarily many complete hyperedges offers us a possibility of having applications in quantum computation. We present three such non-KS NBMMPHs with single complete hyperedges in lower dimensions in Figure 9 and their strings and coordinatizations in Appendix E.

### 2.8. Contextuality—Noncontextuality Dichotomy

Recently Williams and Constantin [81] (p. 2) attempted to set a 3D lower bound for KS sets. Since this is suggested to be helpful in generating contextual sets from noncontextual ones, let us consider the proposal in some detail.

**Figure 10:**
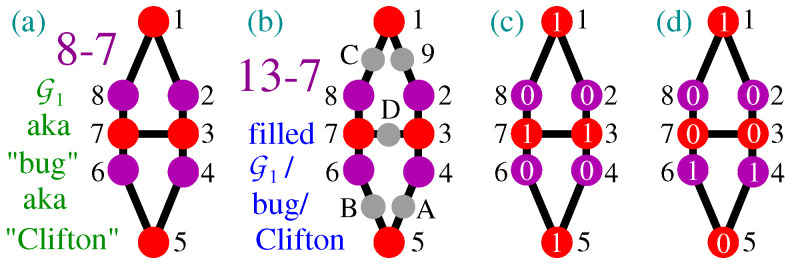
(**a**) 8-7 contextual non-KS bug/Clifton NBMMPH; (**b**) noncontextual 13-7 filled bug/Clifton; (**c**,**d**) two mutually contradictory Clifton-like ‘proofs’: Propositions 1 and 2; see text.

They start with the Clifton hypergraph $G_{1}$ [82]—a.k.a. *bug* [83]—shown in Figure 10a, which is a contextual non-KS NBMMPH. The filled $G_{1}$ shown in Figure 10b is neither a KS nor a non-KS NBMMPH, i.e., it is a noncontextual MMPH.

For this hypergraph $G_{1}$, Clifton [82] himself and also Ramanathan, Rosicka, Horodecki, Pironio, Horodecki and Horodecki [84]; Williams and Constantin [81] (p. 2); and others claim that the following Proposition 1 holds:

**Proposition** **1.** *Vertices* 1 *and* 5 *of $G_{1}$ cannot both be assigned value ‘*1*’.*

Their ‘proof’ runs as follows. Suppose v(1)=v(5)=1. Then the condition (i) of Definition 3 implies that v(2)=v(8)=v(4)=v(6)=0 and condition (ii) implies that v(3)=v(7)=1. However, condition (i) does not allow that, hence a contradiction. See Figure 10c.

Ergo, they seem not to be aware of the fact that the 8-7 $G_{1}$ is contextual and that the contradiction stems from that same contextuality, i.e., that the ‘contradiction’ does not prove Proposition 1—it is merely a consequence of the contextuality. To see this, let us consider the opposite proposition.

**Proposition** **2.** *In $G_{1}$, vertex* 5 *cannot be assigned value ‘*0*’ once vertex* 1 *is assigned value ‘*1*’ and vice versa.*

The ‘proof’ would run as follows. Suppose v(1)=1 and v(5)=0. Then the condition (i) of Definition 3 implies that v(2)=v(8)=0 and v(4)=v(6)=1 and condition (i) implies that v(3)=v(7)=0. However, condition (ii) does not allow that, hence a contradiction. See Figure 10d.

We see that both propositions are wrong. Their ‘contradictions’ stem from our inability to assign 1-0 values to all vertices of a contextual set so as to satisfy the conditions (i) and (ii) of Definition 3.

Proposition 1 led to a number of objectionable results and/or claims in the literature. Among them is Williams and Constantin’s endeavor to find a lower bound for the size of 3D KS MMPH, i.e., a minimal possible one. Let us dwell on it.

**Figure 11:**
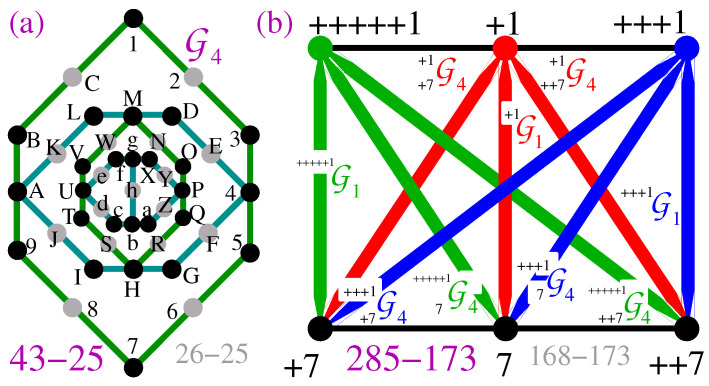
(**a**) $G_{4}$—nested $G_{1}$s [81] (Figure 3). (**b**) ‘168-direction’ set [81] (Figure 4); the top line is the inner triangle and the bottom one is the outer triangle of [81] (Figure 4); note that a clique/triangle in the graph notation is represented by a line in the hypergraph notation; ‘+’s, ‘++’s, … refer to repeated notations of successive Gs for automated processing.

In Section II.C of their paper they construct $G_{4}$—four nested $G_{1}$s—and sketch it in [81] (Figure 3). We give a detailed hypergraph of $G_{4}$ in Figure 11a.

In [81] (p. 2, bottom) Williams and Constantin claim that, following their Proposition 2 (our Proposition 1), U and P of $G_{4}$ cannot both be assigned value 1. But since Proposition 1 does not hold, their claim does not hold either.

Not being aware of that untenability, by means of $G_{4}$s and $G_{1}$s they construct a 168-direction set [81] (p. 2, bottom), whose MMPH version is shown in Figure 11b, and claim that, following their assumption that opposite vertices of $G_{1}$s and of nested $G_{1}$-like shells within $G_{4}$s cannot both be assigned value 1, the 168-direction set is a non-KS NBMMPH. However, since the assumption does not hold, their deduction is not valid, although the 168 NBMMPH *is* a non-KS NBMMPH simply because it contains $G_{1}$, which is a non-KS NBMMPH.

The facts are as follows.

(i) $G_{1}$ 8-7 is a non-KS NBMMPH, $G_{4}$ 26-25 is a non-KS NBMMPH because it contains $G_{1}$s which are non-KS NMMPHs, and the 168-173 MMPH is a non-KS NBMMPH because it contains $G_{1}$s which are non-KS NBMMPHs.
(ii) Neither filled $G_{1}$ 13-7, nor filled $G_{4}$ 43-25, nor filled 168-direction NBMMPH 285-173 are either KS or non-KS NBMMPHs as follows from our programs which are freely available from our repository [85]:
  COMMAND: states01 < 285-173
  OUTPUT: 285-173 admits {0,1} state.
(iii) There is a fundamental difference between the 168-direction NBMMPH and, say, the original Kochen–Specker NBMMPH shown in Figure 2. While both of their stripped versions, 168-173 and 117-118, respectively, are non-KS NBMMPHs and therefore contextual, their filled versions, 285-173 and 192-118, respectively, differ fundamentally—the 285-173 is a noncontextual/classical set, while the 192-118 is a critical KS contextual/quantum set.
(iv) Next, the coordinatization of 285-173 indicated in [81] (Figure 4) is not as simple as presented. A coordinatization of 192-168 (obtained more than 50 years after [30]) requires at least 24 vector components and a computation over several months on a supercomputer [35] (Supplementary Materials). Note that the 192-118 contains 15 filled $G_{1}$s and that all vertices in them have to be different.
(v) The 285-173 contains 3 filled $G_{1}$s and 6 filled $G_{4}$s, and finding vector components and computing all the vertices/vectors are practically unfeasible even on supercomputers. Note that a coordinatization of 168-173 must follow from a coordinatization of 285-173.
(vi) Of the critical non-KS NBMMPH contained in 117-118 and 168-173 sets, some of which are given in Table A1 in Appendix C, only 1% mutually overlap, meaning that the only benefit of the 168-173 set is to be a source of new non-KS NBMMPHs.

Williams and Constantin introduced noncontextual sets [81] (p. 3, Section III.A, Definition 3) and called them ‘*non-KS*’ sets, in contradistinction with the term ‘non-KS’ NBMMPH introduced two years earlier in [86]. Therefore we denote their ‘*non-KS*’ MMPHs by *non-Q* MMPHs. They define their *non-Q* set as follows.

**Definition** **10.** *In $H^{n}$, n≥3, there is a set A of vectors ws pointing to the points on an (n−1)-dim sphere $S^{n-1}$—called a* non-Q*—that admits a valuation map: A→{0,1} such that*

*(i)*    *n(−w)=n(w) for all vectors in A;*
*(ii)*   *$\sum_{i\in I}v\left(w_{i}\right)\leq 1$ for all sets of mutually orthogonal vectors ${\left\{w_{i}\right\}}_{i\in I}$ in A;*
*(iii)*  *$\sum_{i\in I}v\left(w_{i}\right)=1$ for all sets of n mutually orthogonal vectors ${\left\{w_{i}\right\}}_{i\in I}$ in A*

Further, Williams and Constantin [81] (p. 6, Section III.B) put forward:

**Proposition** **3.** *The union of any non-Q set with its antipodal is also a* non-Q *set.*

In effect, this boils down to a claim that a union of any of ‘non-Q’ sets with a coordinatization with its antipodal set with a coordinatization is ‘non-Q’ set with a coordinatization. Let us see whether this holds.

**Figure 12:**
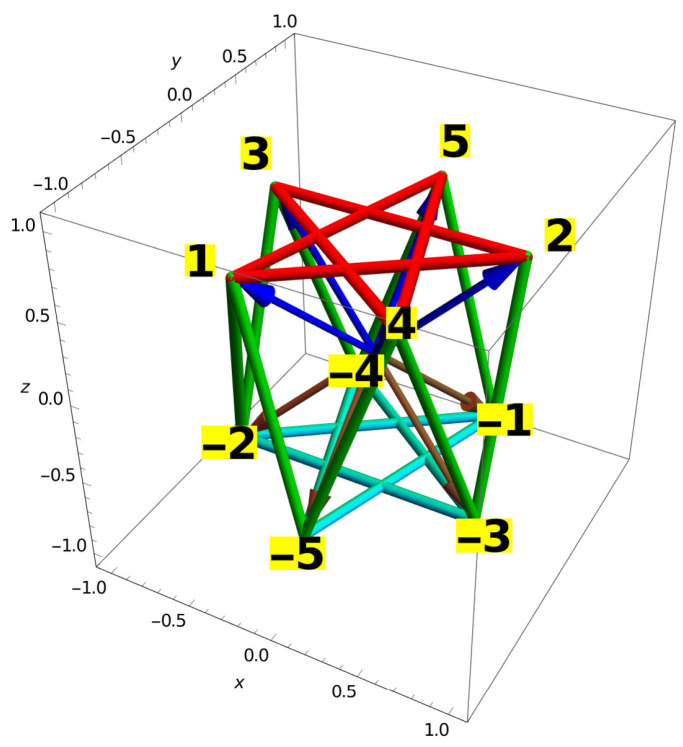
MMPH 10-20 consisting of a double-pentagon and 10 3D (green, upright) hyperedges generated by vector orthogonalities; notice that there are loops of order four (quadrilaterals), e.g., 1-2-(−1)-(−3)-1, which do not allow a coordinatization of the MMPH [33].

When dealing with sets, Williams and Constantin actually deal with vectors themselves, not with hyperedges the vectors determine. So, if we took the upper pentagon of vectors from Figure 12 to be *A*, then the lower pentagon of vectors would be −A.

But the pentagons themselves are formed by the hyperedges, which not only connect the heads of just two mutually orthogonal end vectors but also contain a third vector in between them since they live in the 3D space and since the vertices that define them are also mutually orthogonal—cf. [29] (Figure 5). The fact that we eventually carry out our further possible calculations with data corresponding to only two end vectors does not change the fact that every hyperedge in 3D corresponds to a triple of vectors—the geometry of orthogonality requires three vectors orthogonal to each other to define the hyperedges. Whether we discard data of measurements carried out in one of the three directions does not eliminate the corresponding port of the apparatus nor change the geometry of vectors in 3D. The problem with the proposed construction is that such third vectors cannot have coordinatizations and that therefore the whole construction is inconsistent in spite of the fact that filled *A*s, i.e., pentagons, do have a coordinatization.

The vectors, by their orthogonality, define the dark green hyperedges shown in Figure 12. All the hyperedges together define a hypergraph/set orthogonalities of vectors. But the vectors cannot transfer their coordinatization to the hypergraph in 3D since it contains loops of order four, e.g., 1287 (1,2,−3,−2) [33] (Section 5(x)). Details are given in Appendix D.

Taken together, *A* might be a set of vectors with a coordinatization, that form a contextual hypergraph pentagon with a coordinatization, but the A∪−A is an equivocation—it should be a hypergraph with a coordinatization since both *A* and −A do have a coordinatization, but it cannot be a hypergraph with coordinatization in 3D since the hypergraph with a square loop of hyperedges (quadrilateral) cannot have a coordinatization in 3D.

This brings the validity of the lower bound for the maximal non-Q [81] (p. 6, Equation (24)) into question since its derivation [81] (p. 5) assumes that A∪−A is a noncontextual set with a coordinatization, but that does not hold since we proved above that their pentagon-hypergraph cannot have a coordinatization.

Then they proceed as follows: ‘Hence a lower bound for the size of a KS set can be established by finding the smallest set of directions that any given non-Q set avoids’ and arrive at Equation (29) of [81]. But since one cannot say whether a non-Q set ‘avoids’ KS sets because it might not have a coordinatization, this result might not hold, either. In any case Williams and Constantin have not arrived at any explicit form of a 3D contextual set.

### 2.9. How Abundant Is Contextuality?

In previous sections we searched for applications and generations of contextual sets that are not just proofs of their non-classicality. At the first sight our finding seemed to be not very abundant. So, it is interesting to make use of noncontextuality to approach the problem from the other side to see how often a set of states and measurements admits a classical interpretation. That would give us an answer to the question on how many of sets of quantum states and their measurements are contextual and how many are not. In other words ‘How typical is contextuality?’ [87]. Question: ‘How typical is it to stumble upon contextuality when we randomly sample a finite number of pure states and projective measurements?’ Answer: ‘Using numerical linear programs to test for the existence of a generalized-noncontextual model, we find that contextuality is fairly common: even in experiments with only a modest number of random preparations and measurements, contextuality arises with probability over 99%.’ [87] (pp. 1, 3).

This vividly prompts future research of contextuality unrelated to hidden variable theories.

## 3. Discussion

In this paper we search for possible emergence of contextuality from quantum theory or quantum measurements unrelated to any hidden variable theory quantum experiments might beat. To be more precise, we explore the implications of rejecting the premise that a set of predetermined measurement outcomes constitutes a valid starting point for finding applications of quantum contextuality. On the road to this goal we encounter a number of obstacles. Some of them we bring forward in detail.

We start with an emergence of contextuality which can definitely satisfy our restriction, i.e., which emerges directly from protocols of quantum computation and its error correction procedures and which we elaborate on in Section 2.2. Here we deal with a procedure developed in Ref. [22] (see also [23]) where a Hadamard gate applied on qubit states combined with ancillary qubits in magic states yield non-KS NBMMPH shown in Figure 3a. The obtained MMPH is not critical, hence not minimal, and therefore we generate a KS MMPH which contains it, and from the latter MMPH we obtain a smaller critical MMPH shown in Figure 3b. This procedure of dealing with non-KS contextual sets vs. KS sets has predecessors from more then half century ago, about which there are still widespread misconceptions today. So, we actually start the section with a discussion of misconceptions which boil down to the fact that, e.g., the famous 3D non-KS NBMMPHs like original Kochen–Specker 117-118 or Peres’ 33-40 sets are taken to be the smallest (minimal) in their classes in spite of the evidence that they are not critical sets as pointed out in Figure 2.

The next possible application we consider (In Section 2.3) is pseudo-telepathy games. When a state of one of two entangled particles is determined by a measurement we immediately know what a measurement of the state of the other particle would yield. Someone might be tempted to ascribe telepathic ability to the participants, but since they cannot send information to each other via entanglement, the term pseudo-telepathy was introduced to name the games. Originally the pseudo-telepathy games dealt with KS MMPHs, i.e., they carried out verification of orthogonalities between entangled states belonging to complete bases (hyperedges with *n* vertices, where *n* is the dimension of the MMPH), and they served for disproving hidden variable theories. Recently, games with non-KS NBMMPHs were considered with verification of orthogonalities or non-orthogonalities, and we checked whether they can offer applications beyond invalidating hidden variable theories. Surprisingly we find that the games were limited by a misconceived lower bound of non-KS NBMMPHs containing a certain number of complete bases. We show that various sets of vector components generate corresponding master sets which generate their class of smaller KS MMPHs. In 3D every such KS MMPH contains many vertices contained in only one hyperedge. When we remove them we obtain non-KS NBMMPHs which are predominantly non-critical. The smallest critical 3D non-KS NBMMPH is always smaller than the smallest critical KS, and the lowest number of complete bases of non-KS NBMMPHs is always smaller than the number of complete bases in the smallest KS MMPHs. Hence, to stop simplifying the game at the latter number of complete bases is unfounded. When analyzing approaches we focused on MMPHs generated by complex vectors and found that some of them, due to their symmetry, can be given a 3D representation which offers the readers interactive views from any angle through links of Figure 5 and Figure 6b. Whether the considered versions of the pseudo-telepathy games can have applications beyond disproving hidden variable theories remains to be addressed. We do not find any.

In Section 2.4 we make use of mutual convertibility of contextuality and nonlocality to elaborate on the quantum advantage of quantum over classical shallow circuits, which is due to the quantum nonlocality. This is implicitly another application of quantum contextuality in quantum computation, although its technical details should be translated to a hypergraph language, presumably more general than the MMPH language. In the section we also consider a Hardy-type contextuality, which is equivalent to logical contextuality, and its nonlocality. Its three-path interferometer implementation developed by Holger Hofmann enables weak measurements which are not accessible by direct measurements.

In Section 2.5 we point out that the GLS inequality is violated by arbitrarily many MMPHs and that it is therefore not a noncontextuality inequality and cannot distinguish contextual from noncontextual sets with standard measurements.

Recently proposed quantum cryptography based on a KS protocol is claimed to be ‘protected’ by contextuality. In Section 2.6 we consider its protocol and find that it would be protected by contextuality only if an eavesdropper assumed that Alice and Bob exchange messages organized under predetermined assignments of bits to vertices of a chosen MMPH. But since Eve knows that Alice and Bob’s assignments are quantum she is not going to base her interception on some predetermined hidden-variable model, and therefore Alice and Bob cannot achieve any contextual advantage or ‘protection.’ Yet, the MMPH large alphabet protocol does offer higher security simply because it can draw inputs from a vastly abundant source of MMPHs.

An interesting relationship between generalized complex Hadamard matrices and star-like MMPHs is considered in Section 2.7. With the proviso that complex Hadamard gates will find their applications in quantum computation, Lisoněk’s Theorem 1 yields existence of NBMMPHs for every S-Hadamard matrix of even order *n* which establishes a relation between S-Hadamard matrices and NBMMPHs. The non-KS NBMMPH versions of them are manageable, but details of correlations await elaboration.

In Section 2.8 we consider an attempt to construct a large noncontextual MMPH in 3D and to arrive at a lower bound on size of a contextual MMPH which starts with an incorrect theorem what affects further steps of the procedure. The theorem in question is a well-known Clifton theorem which claims that the opposite vertices of the so-called ‘bug’ (Clifton’s hypergraph) cannot both be assigned value ‘1’. Since we are able to prove an opposite claim the Clifton theorem turns out to be false. The whole construction of a large MMPH which follows this false assumption is instructive, and we present it in some detail with the help of *Mathematica 14.3* calculations and 3D representation shown in Figure 12 since the procedure shows how one can generate MMPHs via a method which differs from those ones supported by existing methods and algorithms.

In Section 2.9 we consider a recent question on how often we stumble upon contextuality when we randomly sample a finite number of pure states and projective measurements, or, how typical contextuality is. And the answer might be ‘with the probability of over 99%’.

Taken together we show that there is only a handful of possible genuinely quantum applications of contextual MMPHs. We find that they require further elaboration and that they have not been properly addressed as of yet because researches have been focused on disproving hidden variable theories and finding smallest sets and games and protocols for them instead of using available sets of any kind, size, and dimension to investigate their further possible usage. This is so far as physics of contextuality is concerned. Finding new methods of generating contextual sets would remain a valuable contribution to the hypergraph theory as a mathematical discipline.

## 4. Methods

The methods we employ to manage quantum contextual sets are based on algorithms and programs developed within the MMPH language, including Shortd, Mmpstrip, One, Mmpsubgraph, Vecfind, and States01, as referenced in [29]. These resources are freely accessible at http://puh.srce.hr/s/Qegixzz2BdjYwFL (accessed on 6 April 2026). MMPHs can be visualized as hypergraph figures, consisting of dots and lines, and can also be represented as strings of ASCII characters. This representation enables the simultaneous processing of billions of MMPHs using supercomputers and clusters. To facilitate this processing, we have developed additional dynamical programs to manage and parallelize tasks involving any number of MMPH vertices and edges. For 3D representations we made use of Blender 5.0 and Mathematica 14.3.

## 5. Conclusions

The field of contextual sets has emerged from an endeavor to invalidate the possibility of hidden variables underlying quantum mechanics. After more than half a century of diligent research and development, automated methods of generation of a plethora of various kinds of sets of arbitrary structure in any dimension have been developed, alongside extensive experimental validation. Therefore, in this paper we investigate whether one can engineer contextual sets from quantum artifacts (models, structures, and/or measurements) or make use of them to arrive at possible quantum applications without a reference to any hidden variable theory. In doing so, we highlighted and resolved some misconceptions that might hinder the achievement of the goal. Formal definitions of quantum engineering of contextual sets and of their quantum application are given in Section 2.1 by Definitions 8 and 9, respectively. A more detailed summary of the achieved results is given in Section 3, but a succinct overview of them is as follows.

(a) In Section 2.2 we analyze a quantum engineering and a quantum application in the field of quantum computation. Quantum engineering consists of a procedure in which Hadamard gates applied on qubit states combined with ancillary qubits in magic states yield contextual non-KS NBMMPHs. We show that they contain smaller non-KS NBMMPHs. Quantum application consists of an application of the obtained non-KS NBMMPHs to error correction algorithms within quantum computation. Open questions are whether there are noncontextual sets that can serve the same purpose; if there are, whether the contextual ones would provide some advantage over them; and whether smaller non-KS NBMMPHs we find correspond to more efficient quantum gates.
(b) In Section 2.3 we consider pseudo-telepathy games. The original protocols based on verification of vertex orthogonalities are just another way of disproving the hidden variable theories. Therefore we consider a recent non-orthogonality pseudo telepathy proposal to see whether it might contribute to quantum computation or some other quantum applications. We start with an analysis of several unsupported premises. First, the validity of Equation (3b) is implicitly assumed, although it is not valid in the considered domain—see Figure 4b. Second, the minimality of the considered MMPH (33-50) is assumed, although it is not critical and therefore not minimal—see Figure 7b. Third, a special role and a ‘minimality’ of the ‘complete bases’ are assumed, although we find MMPHs with smaller complete bases that might serve the purpose. When rectifying these points we find that the proposal in its present form can be used just to disprove hidden variable theories. Yet, if one found quantum dynamics with variable probabilities at gate ports, the inequalities given by Equation (3a,b) would hold and might be employed to gauge, calibrate, and classify sets of quantum systems within the quantum computation procedure. This requires further investigation.
(c) In Section 2.4 we elaborate on possible application of contextuality in quantum computation where a quantum advantage can be achieved via quantum shallow circuits, which is shown to be a consequence of quantum nonlocality, which can be converted to quantum contextuality. Further research on reformulating shallow circuit protocols in the hypergraph language is required. We also consider a Hardy-type contextuality, i.e., logical contextuality, and its nonlocality. Not all Hardy-type contextualities enable their representation via MMPHs, but recently developed three-path interferometer implementation of such a contextuality does. It is based on a particular kind of weak measurements, which enable linking between the wave-like propagation of quantum particles and their localized detection in only one of the output paths. Such weak measurements, not accessible by direct measurements, have applications other than contextual ones. Comparing their contextual with noncontextual properties might help us to improve weak value amplification, e.g., in precision metrology.
(d) In Section 2.5 we point out that the GLS inequality Equation (3b) is violated by standard quantum measurements on standard quantum apparatuses—see Figure 4b. It therefore cannot distinguish between contextual and noncontextual sets and cannot serve for evaluating sets within a standard quantum application. Whether it could serve this purpose within setups with non-standard variable probability distribution over gates remains to be further investigated.
(e) In Section 2.6 we consider quantum cryptography protocol, which is claimed to be ‘protected’ by contextuality. Alice and Bob exchange messages organized by quantum measurement outputs assigned to vertices of a chosen NBMMPH. The protection might be efficient only providing the eavesdropper assumes predetermined assignments of bits to vertices. But in reality she would not carry out such a hidden variable assumption, and consequently contextuality protection would not come to the fore.
(f) In Section 2.7 we consider connection between generalized complex Hadamard matrices and star-like NBMMPHs. We show that every S-Hadamard matrix of even order corresponds to a star-like KS NBMMPH but find that these MMPH are computationally very demanding in higher dimensions. However, we also show that all sub-MMPHs are critical and might establish correlation with truncated S-Hadamard matrices. That requires further elaboration.
(g) In Section 2.8 we consider a 3D noncontextual construct and an attempt to use it to arrive at a lower bound on size of a contextual set. It is marred by the incorrectness of the theorem which the attempt relies on. The theorem in question is the Clifton theorem, which we prove to be false. However, the procedure of building up the construct is very instructive since it shows that geometric designs of hyperedge loops take us to unforeseen orthogonalities, which correspond to hidden hyperedges which ruin coordinatization—see Figure 12. Yet, the whole construction might be interesting for possible generations of contextual sets without any reference to classical sets with predetermined evaluations of their elements.
(h) In Section 2.9 we consider a recent question on how often we stumble upon contextuality in random quantum setups and measurements. The answer ‘with probability over 99%’ prompts further research of contextuality per se.
(i) In several sections, we found out that particular approaches went astray due to a lack of adherence to the syntax of the quantum contextuality language. A unification of notations, language, and methods should be a conditio sine qua non. AI should be programmed to learn directly from scientific papers and to distill all results in a lingua franca of contextuality and hypergraphs.

## Data Availability

The data presented in this study are openly available in Puh, Croatia at http://puh.srce.hr/s/Qegixzz2BdjYwFL (accessed on 6 April 2026), reference number [85].

## Abbreviations

The following abbreviations are used in the manuscript:

MMPH-dimension	McKay–Megill–Pavičić hypergraph dimension (Definition 1)
MMPH	McKay–Megill–Pavičić hypergraph (Definition 2)
NBMMPH	Non-binary McKay–Megill–Pavičić hypergraph (Definition 3)
BMMPH	Binary McKay–Megill–Pavičić hypergraph (Definition 4)
KS	Kochen–Specker (Lemma 1)
non-KS	Non-Kochen–Specker (Lemma 2)

## Appendix A. 3D KS MMPHs Generated from Simple Vector Components

Today, we can generate arbitrarily many MMPHs in any dimension from simple vector components [38] (Table 2) via an automated procedure in no CPU time. In 3D, vector components {0,±1,±2,5} yield the 97-64 class with 20 critical MMPHs, which include Bub–Schütte’s set; 9 non-isomorphic 51-37 MMPHs, one of which is Conway-Kochen’s set; a 53-38; 8 54-39; and a 55-40 [35] (Table I). $\left\{0,\pm 1,\pm \sqrt{2},3\right\}$ yields the 81-52 master, which contains a single critical set—Peres’ set; a set of 24 vector components explicitly given for the first time in [35] (Supplementary Materials, p. 3) yields the original KS set; and $\left\{0,\pm \omega ,2\omega ,\pm \omega^{2},2\omega^{2}\right\}$, where $\omega =e^{2\pi i/3}=\left(-1+i\sqrt{3}\right)/2$, yield the 169-120 class which contains 514 critical sets, the smallest of which is the 69-50 explicitly given in [29] (Figure 10e, p. 55).

In the past, the authors of the first 3D KS sets dropped vertices/vectors belonging to only one hyperedge (multiplicity 1; gray dots) just because the remaining vertices suffice for simpler KS proofs. For instance: 49-36: Bub: ‘Removing…16 rays …that occur in only one orthogonal triple …from the 49 rays yields …[a] set of 33 rays’ [88].

## Appendix B. Some Pseudo-Telepathy Game NBMMPHs

As for the generation of masters from complex numbers, apart from $\left\{0,\pm \omega ,2\omega ,\pm \omega^{2},2\omega^{2}\right\}$ we used of in [29,34], here we use $\left\{0,\pm 1,\pm \omega ,2\omega ,\pm \omega^{2}\right\}$, which yield the 204-149 master containing over 10,000 critical KS MMPHs (69-50 the smallest, 130-95 the largest), as well as $\left\{0,\pm 1,\pm \omega ,2\omega ,2\omega^{2}\right\}$, which give the 163-102 master, which contains only one critical set: the 69-50 set. The 69-50 sets contained in any of those masters are isomorphic.

## Appendix C. Comparison of 117-118 and 168-173 NBMMPHs

**Table A1:** Critical non-KS hypergraphs contained in 117-118 and 168-173, obtained via our program States01 on a supercomputer. Among 100,000 hypergraphs obtained from each of them, there are 229 and 663 non-isomorphic ones, respectively, the smallest and the largest of which are shown below.

117-118			168-173		
Vertices	Hyperedges	No.	Vertices	Hyperedges	No.
8	7	1	8	7	1
14	10	1	-	-	-
-	-	-	14	11	2
15	11	1	15	11	5
17	12	1	-	-	-
-	-	-	16	13	2
-	-	-	17	13	4
18	13	1	18	13	6
…	…	…	…	…	…
44	32	5	44	32	2
…	…	…	…	…	…
-	-	-	43	34	4
47 Max	34 Max	1	-	-	-
…	…	…	…	…	…
-	-	-	64	47	3
-	-	-	67 Max	49 Max	2

## Appendix D. Union of Antipodal Non-Q Sets Might Not Have a Coordinatization

Antipodal vectors −3, −2, … do have a coordinatization: $-3=(\left(\sqrt{3}-\sqrt{5}/2,\sqrt{1+1/\sqrt{5}}/2,-5^{-1/4}\right)$, $-2=(-1/\sqrt{2},-\sqrt{1/2-1/\sqrt{5}},-5^{-1/4})$, …, but they cannot build a hypergraph together with 3, 2, …Third vectors that should be present in the middle of the hyperedges/triples (orthogonal to two end vectors)—cf. Figure 5 in [29]—simply do not exist—vertices do not have a coordinatization.

When we take the negative vectors −1, …, −5 from Figure 12 to mean just hypergraph vertices and rename them so as to read 6, …, A, we obtain the string which represents the double-pentagon together with the vertical green edges (with 1, …, 5 also taken to be just vertices and not any more vectors):

**10-20** 12,23,34,45,51,17,1A,26,28,37,39,48,4A,56,59,67,78,89,9A,A6.

It is obviously a non-KS set, i.e., a contextual set:

COMMAND: states01 −1 < 10-20
  OUTPUT: 10-20 fails (admits no {0,1}) state)
  because it contains pentagons which are contextual.

If we filled up both pentagons and upright dark green hyperedges, we would obtain the following 30-20 MMPH: 1B2,2C3,3D4,4E5,5F1,1G7,1HA,2I6,2J8,3K7,3L9,4M8,4NA,5O6,5P9,s6Q7,7R8,8S9,9TA,AU6. which is noncontextual (non-Q):

COMMAND: states01 −1 < 30-20
  OUTPUT: (30-20) passes (admits {0,1} state) but it does not have a real coordinatization.

## Appendix E. NBMMPH Strings Referred to in the Paper

**69-50** 123,145,267,389,9YA,5ZA,4aB,6bB,7cC,8dC,5eD,6fD,8gD,4hC,7iA,9jB,1EF,2GH,3IJ,KkF, KlJ,KmH,LnE,LoG,LpJ,MqH,MrI,MsE,NtF,NuG,NvI,1OP,2QR,3ST,UwO,UxT,UyR,VzP,V!Q,V"T,W#R,W$S, W%P,X&O,X’Q,X(S,BLV,CMW,AKU,DNX. 1=(0,0,1), 2=(0,1,0), 3=(1,0,0), 4=(1,−1,0), 5=(1,1,0), 6=(1,0,−1), 7=(1,0,1), 8=(0,1,1), 9=(0,1,−1), A=(−1,1,1), B=(1,1,1), C=(1,1,−1), D=(1,−1,1), E=(ω,−1,0), F=(ω,1,0), G=(1,0,−ω), H=(1,0,ω), I=(0,ω,1), J=(0,ω,−1), K=(−1,$\omega^{2}$,ω), L=(1,$\omega^{2}$,ω), M=(1,$\omega^{2}$,−ω), N=(1,$-\omega^{2}$,ω), O=(1,ω,0), P=(1,−ω,0), Q=(ω,0,−1), R=(ω,0,1), S=(0,1,ω), T=(0,1,−ω), U=(−1,ω,$\omega^{2}$), V=(1,ω,$\omega^{2}$), W=(1,ω,$-\omega^{2}$), X=(1,−ω,$\omega^{2}$), Y=(2ω,ω,ω), Z=(ω,−ω,2ω), a=(−ω,−ω,2ω), b=(−ω,2ω,−ω), c=(−ω,2ω,ω), d=(2ω,−ω,ω), e=(−ω,ω,2ω), f=(ω,2ω,ω), g=(2ω,ω,−ω), h=(ω,ω,2ω), i=(ω,2ω,−ω), j=(2ω,−ω,−ω), k=(1,$-\omega^{2}$,2ω), l=(2ω,1,$\omega^{2}$), m=($\omega^{2}$,2ω,−1), n=(−1,$-\omega^{2}$,2ω), o=($-\omega^{2}$,2ω,−1), p=(2ω,−1,$-\omega^{2}$), q=($-\omega^{2}$,2ω,1), r=(2ω,−1,$\omega^{2}$), s=(1,$\omega^{2}$,2ω), t=(−1,$\omega^{2}$,2ω), u=($\omega^{2}$,2ω,1), v=(2ω,1,$-\omega^{2}$), w=($\omega^{2}$,−1,2ω), x=(2ω,$\omega^{2}$,1), y=(1,2ω,$-\omega^{2}$), z=($-\omega^{2}$,−1,2ω), !=(−1,2ω,$-\omega^{2}$), "=(2ω,$-\omega^{2}$,−1), #=(−1,2ω,$\omega^{2}$), $=(2ω,$-\omega^{2}$,1), %=($\omega^{2}$,1,2ω), &=($-\omega^{2}$,1,2ω), ’=(1,2ω,$\omega^{2}$), (=(2ω,$\omega^{2}$,−1)

**85-62** 123,456,789,9e6,9f3,AgB,ChD,EiB,FjD,GHA,IJC,KJE,LHF,MND,OPB,QRJ,STH,UV8,WX8, Pk9,Nl9,Ym7,Zn7,Go6,Ip3,aqE,brF,csA,dtC,Ku5,Lv2,TwN,RxP,ZyR,ZzT, Q!4,S"1,V#5,X$2,X%4,V&1, c’W,d(U,a)W,b*U,dcY,baZ,JH8,a-G,b/I,M:G,O;I,c<K,d=L, M>K,O?L,Q@N,S[P,Y\Q,Y]S,VˆO,X_M. 1=(1,−1,ω), 2=(1,−ω,1), 3=(ω,−1,1), 4=(1,ω,−1), 5=(1,1,−ω), 6=(ω,1,−1), 7=(0,1,−1), 8=(1,0,0), 9=(0,1,1), A=(ω,1,0), B=(1,$-\omega^{2}$,ω), C=(ω,0,1), D=(1,ω,$-\omega^{2}$),E=(1,0,−ω),F=(1,−ω,0),G=(ω,−1,0),H=(0,0,1),I=(ω,0,−1),J=(0,1,0),K=(1,0,ω), L=(1,ω,0),M=(1,$\omega^{2}$,−ω),N=(1,1,−1),O=(1,−ω,$\omega^{2}$),P=(1,−1,1),Q=(1,0,1),R=(1,0,−1),S=(1,1,0), T=(1,−1,0), U=(0,1,−ω), V=(0,1,ω), W=(0,ω,−1), X=(0,ω,1), Y=(−1,1,1), Z=(1,1,1), a=(1,$\omega^{2}$,ω), b=(1,ω,$\omega^{2}$), c=(−1,$\omega^{2}$,ω), d=(−1,ω,$\omega^{2}$), e=(2ω,−1,1), f=(2ω,1,−1), g=(−1,$\omega^{2}$,2ω), h=(−1,2ω,$\omega^{2}$), i=($\omega^{2}$,2ω,1), j=($\omega^{2}$,1,2ω), k=(2ω,ω,−ω), l=(2ω,−ω,ω), m=(2ω,ω,ω), n=(2ω,−ω,−ω), o=($\omega^{2}$,ω,2ω), p=($\omega^{2}$,2ω,ω), q=($-\omega^{2}$,2ω,−1), r=($-\omega^{2}$,−1,2ω), s=(1,$-\omega^{2}$,2ω), t=(1,2ω,$-\omega^{2}$), u=(−ω,2ω,$\omega^{2}$), v=(−ω,$\omega^{2}$,2ω), w=(ω,ω,2ω), x=(ω,2ω,ω), y=(−ω,2ω,−ω), z=(−ω,−ω,2ω), !=(−1,2ω,1), "=(−1,1,2ω), #=(2ω,−ω,$\omega^{2}$), $=(2ω,$\omega^{2}$,−ω), %=(2ω,$-\omega^{2}$,ω), &=(2ω,ω,$-\omega^{2}$), ’=(2ω,1,$\omega^{2}$), (=(2ω,$\omega^{2}$,1), )=(2ω,−1,$-\omega^{2}$), *=(2ω,$-\omega^{2}$,−1), -=(−1,$-\omega^{2}$,2ω), /=(−1,2ω,$-\omega^{2}$), :=(1,$\omega^{2}$,2ω), ;=(1,2ω,$\omega^{2}$), <=($\omega^{2}$,2ω,−1), ==($\omega^{2}$,−1,2ω), >=($-\omega^{2}$,2ω,1), ?=($-\omega^{2}$,1,2ω), @=(−ω, 2ω,ω), [=(−ω,ω,2ω), \=(ω,2ω,−ω), ]=(ω,−ω,2ω), =(2ω,$\omega^{2}$,−1), _=(2ω,−1,$\omega^{2}$)

**152-71** Coordinatization. 1=(0,0,0,1), 2=(0,0,1,0), 3=(0,1,0,0), 4=(1,0,0,0), 5=(0,1,1,0), 6=(0,−1,1,2), 7=(0,1,−1,1), 8=(1,0,0,1), 9=(1,0,0,−1), A=(0,1,−1,0), B=(1,1,−1,−1), C=(1,−1,1,−1), D=(1,1,1,1), E=(1,−1,−1,1), F=(0,0,1,1), G=(2,0,1,−1), H=(1,3,−1,1), I=(0,0,1,−1), J=(1,1,0,0), K=(1,−1,0,0), L=(0,1,0,−1), M=(1,0,1,0), N=(0,1,0,1), O=(1,0,−1,0), P=(1,1,1,−1), Q=(1,−1,1,1), R=(−1,1,1,1), S=(1,1,−1,1), T=(2,3,−3,0), U=(3,−1,1,0), V=(2,3,3,0), W=(−3,1,1,0), X=(1,−1,1,0), Y=(−1,1,2,0), Z=(1,1,3,0), a=(3,3,−2,0), b=(1,1,−1,0), c=(−1,2,1,0), d=(1,1,1,0), e=(1,−2,1,0), f)=(1,3,0,−1), g=(−3,2,0,3), h=(1,3,0,1), i=(3,−2,0,3), j=(1,−1,0,3), k=(−3,3,0,2), l=(1,1,0,3), m=(3,3,0,−2), n=(1,1,0,1), o=(−2,1,0,1), p=(1,1,0,−1), q=(2,−1,0,1), r=(1,0,3,−1), s=(−3,0,2,3), t=(1,0,3,1), u=(3,0,−2,3), v=(1,0,1,−1), w=(2,0,−1,1), x=(1,0,1,1), y=(−2,0,1,1), z=(1,0,−1,1), !=(−1,0,1,2), "=(1,0,1,3), #=(3,0,3,−2), $=(0,1,1,1), %=(0,1,1,−2), &=(0,1,1,−1), ’=(0,2,−1,1), (=(0,−1,1,1), )=(0,2,1,1), *=(0,1,3,1), -=(0,3,−2,3), /=(0,1,3,−1), :=(0,−3,2,3), ;=(1,−2,2,−3), <=(5,−1,1,3), ==(1,−1,1,3), >=(2,1,−1,0), ?=(0,1,−1,2), @=(3,1,−1,−1), [=(1,1,−1,−3), \=(2,−1,1,0), ]=(1,1,3,−1), ˆ=(−1,2,0,1), _=(−1,1,3,1), ‘=(1,2,0,−1), .=(−1,0,2,1), |=(1,−3,1,−1), =(1,0,2,−1), ∼=(1,3,−1,−1), +1=(1,1,−3,1), +2=(1,−2,0,1), +3=(1,−1,3,1), +4=(1,2,0,1), +5=(1,0,−2,1), +6=(1,3,1,1), +7=(1,0,2,1), +8=(1,−3,−1,1), +9=(1,−2,−2,3), +A=(5,−1,−1,−3), +B=(1,2,2,3), +C=(5,1,1,−3), +D=(0,1,1,2), +E=(3,−1,−1,1), +F=(1,−1,−1,3), +G=(2,1,1,0), +H=(1,3,2,2), +I=(5,−3,1,1), +J=(1,1,−1,3), +K=(1,1,2,0), +L=(1,0,−1,2), +M=(−1,3,1,1), +N=(0,1,2,−1), +O=(3,−1,1,1), +P=(1,−3,−2,2), +Q=(5,3,−1,1), +R=(1,−1,3,5), +S=(2,−2,−3,1), +T=(0,1,2,1), +U=(3,1,−1,1), +V=(1,0,1,2), +W=(1,3,1,−1), +X=(1,3,−2,−2), +Y=(5,−3,−1,−1), +Z=(1,1,1,−3), +a=(1,1,−2,0), +b=(0,1,−2,1), +c=(−3,1,1,1), +d=(1,0,1,−2), +e=(1,−3,1,1), +f=(1,−1,5,3), +g=(2,−2,1,−3), +h=(−1,1,1,3), +i=(1,2,−1,0), +j=(0,−1,2,1), +k=(3,1,1,−1), +l=(1,3,−2,2), +m=(5,−3,−1,1), +n=(0,2,1,−1), +o=(3,−1,1,−1), +p=(−1,3,2,2), +q=(5,3,−1,−1), +r=(0,−2,1,1), +s=(3,1,1,1), +t=(−1,1,0,2), +u=(1,−1,−3,1), +v=(1,−1,0,2), +w=(1,−1,3,−1), +x=(1,1,0,2), +y=(1,1,−3,−1), +z=(1,1,0,−2), +!=(1,1,3,1)

**11-7** 123456,67,738,859,92A,A4B,B1. 1=(0,1,0,0,0,0), 2=(0,0,0,0,0,1), 3=(0,0,0,0,1,0), 4=(0,0,0,1,0,0), 5=(0,0,1,0,0,0), 6=(1,0,0,0,0,0), 7=(0,0,0,1,0,−1), 8=(0,0,0,1,0,1), 9=(0,1,0,0,1,0), A=(0,1,0,0,−1,0), B=(0,0,1,0,0,1)

**15-9** 12345678,89,94A,A7B,B3C,C6D,D2E,E5F,F1. 1=(0,1,0,0,0,0,0,0), 2=(0,0,0,0,0,0,0,1),3=(0,0,0,0,0,0,1,0),4=(0,0,0,0,0,1,0,0),5=(0,0,0,0,1,0,0,0),6=(0,0,0,1,0,0,0,0), 7=(0,0,1,0,0,0,0,0),8=(1,0,0,0,0,0,0,0),9=(0,0,0,0,0,0,1,−1),A=(0,0,0,0,0,0,1,1), B=(0,0,0,0,1,1,0,0), C=(0,0,0,0,1,−1,0,0), D=(0,0,0,0,1,1,1,0), E=(0,0,0,0,0,1,−1,0), F=(0,0,0,0,0,1,1,0)

**19−11**   123456789A,AB,B5C,C9D,D4E,E8F,F3G,G7H,H2I,I6J,J1. 1=(0,1,0,0,0,0,0,0,0,0), 2=(0,0,0,0,0,0,0,0,0,1), 3=(0,0,0,0,0,0,0,0,1,0), 4=(0,0,0,0,0,0,0,1,0,0), 5=(0,0,0,0,0,0,1,0,0,0), 6=(0,0,0,0,0,1,0,0,0,0), 7=(0,0,0,0,1,0,0,0,0,0), 8=(0,0,0,1,0,0,0,0,0,0), 9=(0,0,1,0,0,0,0,0,0,0), A=(1,0,0,0,0,0,0,0,0,0), B=(0,0,0,0,0,0,0,1,1,−1), C=(0,0,0,0,0,0,0,0,1,1), D=(0,0,0,0,0,0,0,0,1,−1), E=(0,0,0,0,0,0,1,0,1,1), F=(0,0,0,0,0,0,1,0,0,−1), G=(0,0,0,0,0,0,1,0,0,1), H=(0,0,0,0,0,0,0,1,1,0), I=(0,0,0,0,0,0,0,1,−1,0), J=(0,0,0,0,0,0,0,1,1,1)
